# A case report of thrombotic complete obstruction of the ascending aorta as a complication of Venoarterial extracorporeal membrane oxygenation support: steps to prevent thrombosis

**DOI:** 10.1186/s13019-020-01239-3

**Published:** 2020-07-23

**Authors:** Tasuku Nishihara, Natsuko Kudamatsu, Taisuke Hamada, Yukihiro Nakata, Waichi Yamamoto, Hideyuki Nandate, Kenji Namiguchi, Takashi Nishimura, Hironori Izutani, Toshihiro Yorozuya

**Affiliations:** 1 Department of Anesthesia and Perioperative Medicine, Ehime University Graduate School of Medicine, Toon, Ehime 791-0295 Japan; 2 Department of Cardiovascular and Thoracic Surgery, Ehime University Graduate School of Medicine, Toon, Ehime Japan

**Keywords:** Venoarterial extracorporeal membrane oxygenation, Intra-aortic thrombus, Impella®

## Abstract

**Background:**

Venoarterial extracorporeal membrane oxygenation (VA-ECMO) is an essential device in the field of emergency and intensive-care medicine. However, long-term use of VA-ECMO has various severe complications, including thrombosis.

**Case presentation:**

A 60-year-old man underwent his third aortic root replacement using a homograft because of infectious endocarditis. Although the operation was difficult because of severe adhesion caused by the two previous interventions, aortic root replacement using a homograft was performed. At the time of withdrawal from cardiopulmonary bypass, the maintenance of hemodynamics was difficult because of bleeding from the surgical site, leading to hypovolemic shock. Cardiac function subsequently deteriorated; therefore, VA-ECMO was established and the operation was finished. Three days later, thrombus was formed inside the homograft and completely occluded ascending aorta. Evacuation of hematoma was performed, however, cardiac function was not ameliorated. Eventually, the patient had brain infarction and died. To prevent thrombus formation in very severe low cardiac output cases under VA-ECMO management after surgery, to prevent the stagnation of the blood flow from VA-ECMO will be necessary because anticoagulant therapy will be difficult. Impella ventricular assist device which is recently used widely generates anterograde blood flow and effectively prevents stagnation.

**Conclusions:**

To prevent thrombus formation in cases of very severe low cardiac output, Impella® should be combinatorially introduced from the beginning of VA-ECMO establishment to prevent thrombosis.

## Background

Venoarterial extracorporeal membrane oxygenation (VA-ECMO) is used to manage circulatory failure, such as severe heart failure and pulmonary embolism. The benefit of VA-ECMO is the maintenance of oxygenation and circulation, which are essential in the field of emergency medicine and intensive-care medicine. Conversely, long-term VA-ECMO support has various severe complications, such as bleeding, infection, thrombosis, and related organ infarction (including brain infarction) [1]. In particular, thrombosis subsequently causes thromboembolism, leading to death. The survival rate of cases with a ventricular thrombus is only 2 out of 12 cases [2]. Therefore, the prevention of thrombus formation is an important issue in ECMO management.

In this report, we describe a case of complete aortic obstruction caused by a massive intrahomograft thrombus during VA-ECMO management and discuss the steps that can be used to prevent thrombotic complications.

## Case presentation

**Fig. 1:**
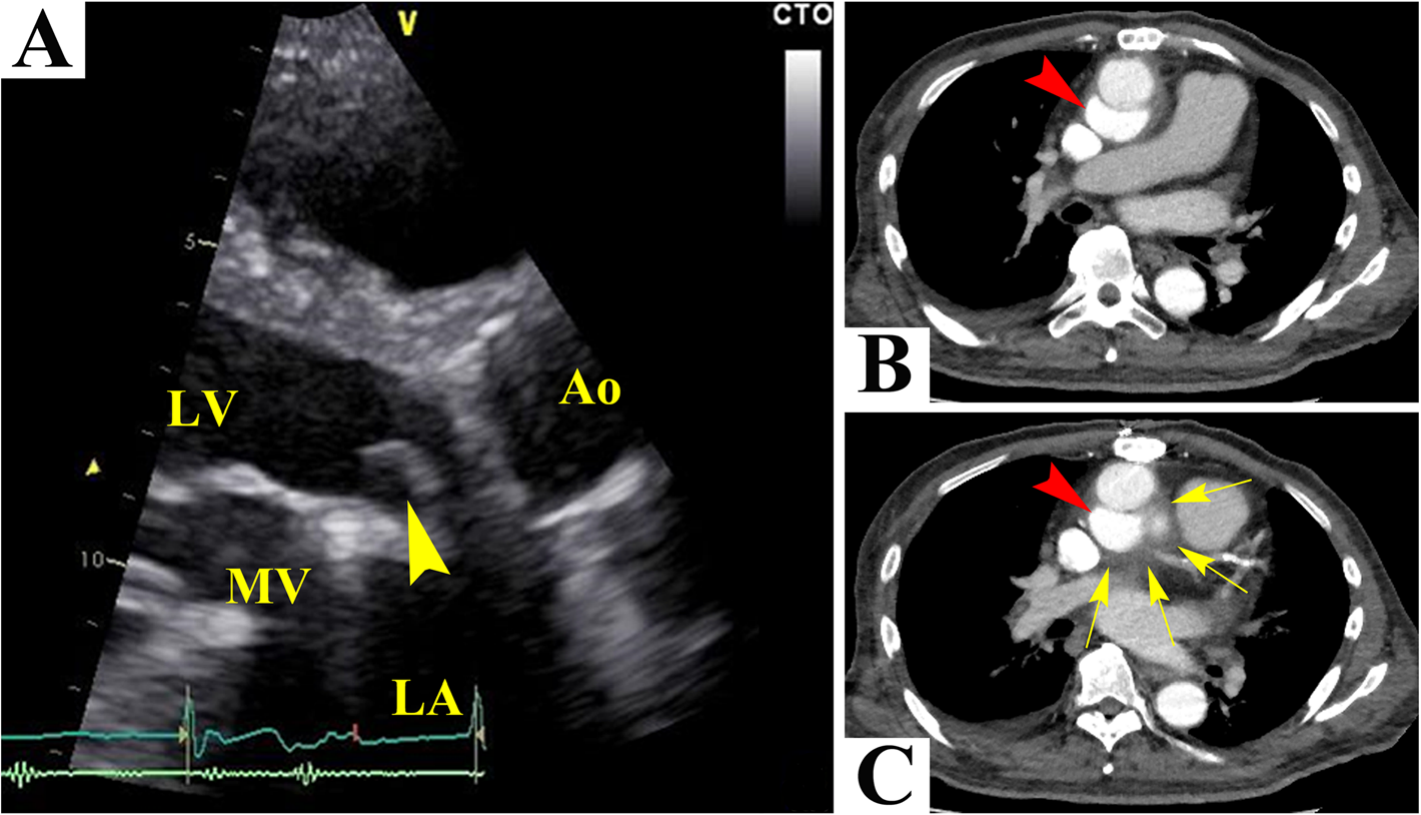
Preoperative examinations. Echocardiography showed infectious endocarditis (IE) in the aortic valve (**a**, arrowhead). Computed tomography revealed a false aneurysm in the dorsum of the artificial vessel (**b**, **c**, arrowheads) and the rise of fat tissue in the mediastinum, indicating mediastinitis (**c**, arrows)

A 60-year-old man was scheduled to undergo his third aortic root replacement using a homograft because of infectious endocarditis in the aortic valve. At the age of 58, he underwent Bentall operation, mitral valve replacement, and Maze operation because of aortic regurgitation, mitral regurgitation, and atrial fibrillation. Two months later, because infection with methicillin-resistant coagulase-negative staphylococci in the artificial vessel was indicated, a second aortic root replacement surgery with coronary artery bypass grafting surgery (CABG) were performed. This time, because he developed a slight fever that persisted for about 3 weeks, he visited our hospital. Infectious endocarditis (IE) in the aortic valve (Fig. 1a), a false aneurysm in the dorsum of the artificial vessel (Fig. 1b, c), and mediastinitis (Fig. 1c) were indicated by echocardiography and computed tomography. As the infection of the artificial vessel was repetitive, a third aortic root replacement using a homograft and CABG were scheduled.

During the hospitalization before the surgery, administration of dopamine, dobutamine, and isoproterenol was started as his circulation dynamics became unstable because of complete atrioventricular block. The examination of blood data before the operation is shown in Supplementary Table 1. On the operation day, anesthesia was induced with a support of catecholamine, followed by the establishment of a cardiopulmonary bypass (CPB) from the femoral artery and vein before starting the operation. Although the operation was difficult and accompanied with bleeding because of severe adhesion attributed to the two previous operations, aortic root replacement using a homograft and CABG were performed as planned; two saphenous vein grafts were connected from the aorta to the left anterior descending artery and right coronary artery. At the time of withdrawal from CPB, we had difficulty in maintaining hemodynamics because of bleeding from the surgical site, which led to hypovolemic shock. Subsequently, his cardiac function deteriorated. Therefore, VA-ECMO was established and the operation was finished.

After the operation, mechanical ventilation and VA-ECMO support were continued in the intensive-care unit. The bleeding from the drains continued. His activated clotting time was in the range of 160 to 180 s without the use of heparin. At postoperation day 1 (POD1), echocardiogram demonstrated cardiac hypofunction, but thrombi were not detected in the right and left atria and ventricles. Improvement of cardiac function was not obtained, pulse pressure decreased gradually, and central venous pressure increased gradually. At POD3, an intrathoracic and pericardial hematoma was suspected and evacuation of hematoma was performed for cardiac tamponade.

**Fig. 2:**
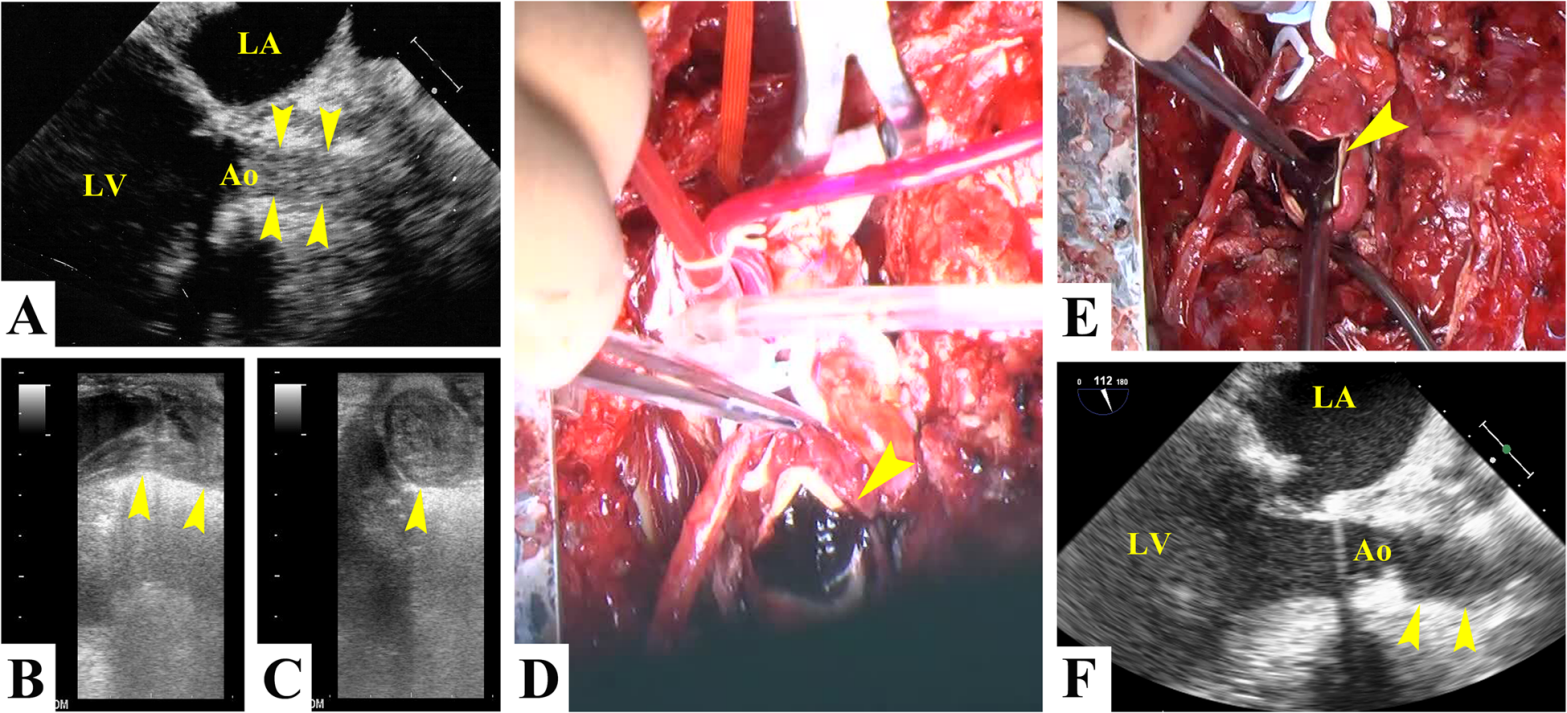
Intraoperative findings. The visualization of the ascending aorta by transesophageal echocardiography was unclear (**a**, arrowheads). Direct echography in the ascending aorta revealed complete obstruction of the homograft by a thrombus (**b**, **c**, **d**). The thrombus located inside the homograft was removed (**e**, **f**)

The hematoma in the thoracic cavity and pericardium was removed by operation. However, cardiac function was not ameliorated. As the visualization of the ascending aorta by transesophageal echocardiography was unclear (Fig. 2a), direct echography in the ascending aorta was performed by the operator to observe the inside of the homograft. The homograft was completely occluded by a thrombus (Fig. 2b, c, d), which was about 4 cm long and was located at the origin of the ascending aorta; however, as the surgeon confirmed the bypass graft patent, the entries of the coronary arteries were intact because entries of the coronary arteries were moved to peripheral side by CABG operation. The thrombus inside the homograft was removed (Fig. 2e, f) and the operation was finished.

After the operation, the pulse pressure was slightly improved but cardiac function was not ameliorated. The patient had brain infarction at POD3 and died at POD5.

## Discussion and conclusions

The reported incidences of complications during VA-ECMO management are 24% of cannulation-related injuries, 20% of bleeding, 11% of left ventricular distention, and 4% of thromboembolic complication [3]. The reported cases of thrombosis are mainly ventricular thrombosis, and intra-aortic thrombosis is extremely rare, with only a few cases reported in the literature [4–7].

In this case, the ascending aorta was completely occluded by the thrombus. Inflammation after the operation may be a possible cause of thrombus formation. Infection as the pathophysiology of the original disease, IE, may also be associated with this complication. However, the major contributor to thrombus formation in this case would be that the movement of the aortic valve was not observed because of severe low cardiac output. In the absence of anterograde blood flow from the ventricle, the aortic valve becomes a dead end for the retrograde blood flow from VA-ECMO. In general, the blood flow may be released from the coronary arteries; however, in this case, the entries of the coronary arteries were moved to the peripheral side by the CABG operation. As a result, the ascending aorta became a blind pouch. The blood flow in the blind pouch stagnated, leading to thrombus formation. Therefore, chronological assessment of cardiac function and exploratory assessment of the thrombus not only in the ventricle, but also in the ascending aorta, are necessary especially in cases of low cardiac output after CABG operation under VA-ECMO management.

Takei et al. reported that the fundamental approach to preventing thrombus formation in the left ventricle is anticoagulant therapy and prevention of left ventricular distension. Thus, they suggested that the placement of a left ventricular assist device (LVAD) at the early stage is an effective option in this setting [8]. In the present case, we hesitated to administer anticoagulant therapy because the bleeding continued after the operation. LVAD implantation may be an option to prevent left ventricular distention; however, LVAD implantation is invasive. Recently, the Impella ventricular assist device (Impella®; Abiomed, Danvers, MA) became used widely for the management of severe low cardiac output cases. Impella® is less invasive as it can be approached by a catheter, which should facilitate its selection vs. LVAD. In fact, left ventricular distention during VA-ECMO management is recognized as a severe problem, [9] and Impella® is reportedly able to unload the left ventricle effectively and provide the heart with real rest [10]. Moreover, the combinatorial use of Impella® with VA-ECMO has been attempted and its superiority in the recovery of cardiac function and mortality compared with VA-ECMO management alone has been recognized [11–13]. Furthermore, Impella® provides anterograde blood flow forcibly and prevents stagnation, which may lead to the prevention of thrombus formation [14]. In this case, the combinatorial use of Impella® should have been considered at the same time with VA-ECMO establishment as the cardiac function was poor, although attention should be paid to the anticoagulant effect of the purge system.

Conclusively, in cases of very severe low cardiac output under VA-ECMO management, we should pay attention to the formation of thrombi not only in the cardiac cavity, but also in the ascending aorta. To prevent thrombus formation in these cases, Impella® should be combinatorially introduced from the beginning of ECMO establishment as another considerable therapeutic option.

## Supplementary information

**Additional file 1: Table S1.** Results of blood examination over time.

## Data Availability

All data generated or analysed during this study are included in this published article.

## Abbreviations

VA-ECMO: Venoarterial extracorporeal membrane oxygenation
CABG: Coronary artery bypass grafting surgery
IE: Infectious endocarditis
CPB: Cardiopulmonary bypass
POD: Post operation day
LVAD: Left ventricular assist device
